# Chronic infection with *Chlamydia pneumoniae* in asthma: a type-2 low infection related phenotype

**DOI:** 10.1186/s12931-021-01635-w

**Published:** 2021-02-26

**Authors:** Doriane Calmes, Pascale Huynen, Virginie Paulus, Monique Henket, Françoise Guissard, Catherine Moermans, Renaud Louis, Florence Schleich

**Affiliations:** 1 Respiratory Medicine, University Hospital of Liege, CHU Sart-Tilman B35, GIGA I3 Lab, Liège, Belgium; 2 Clinical Microbiology, University Hospital of Liege, CHU Sart-Tilman B35, Liège, Belgium

**Keywords:** *Chlamydia pneumoniae*, Asthma, Phenotypes, Neutrophils, Sputum, Macrolides

## Abstract

**Background:**

*Chlamydia pneumoniae* and *Mycoplasma pneumoniae* have been implicated in the pathogenesis of asthma and are responsible for chronic inflammation when host immune system fails to eradicate the bacteria.

**Method:**

We performed a prospective study on 410 patients who underwent a visit at the asthma clinic of CHU of Liege between June 2016 and June 2018 with serology testing for *C. pneumoniae* and *M. pneumoniae*.

**Results:**

65% of our asthmatic population had serum IgA and/or IgG towards *C. pneumoniae*, while only 12.6% had IgM and/or IgG against *M. pneumoniae*. Compared to seronegative asthmatics, asthmatics with IgA+ and IgG+ against *C. pneumoniae* were more often male and older with a higher proportion of patients with smoking history. They received higher doses of inhaled corticosteroids (ICS) and displayed lower FEV_1_/FVC ratio, higher RV/TLC ratio and lower conductance. They had higher levels of fibrinogen, though in the normal range and had lower sputum eosinophil counts. Patients with IgA− and IgG+ against *C. pneumoniae* were older and had higher blood monocyte counts and alpha-1-antitrypsin levels as compared to seronegative patients. Patients with IgM and/or IgG towards *M. pneumoniae* were more often males than seronegative asthmatics. In a subpopulation of 14 neutrophilic asthmatics with *Chlamydia pneumoniae* IgA + /IgG + treated with macrolides, we found a significant decrease in blood neutrophils and normalization of sputum neutrophil count but no effect on asthma quality of life and exacerbations.

**Conclusion:**

Positive Chlamydia serologic test is more common than positive Mycoplasma serology. Asthmatics with IgA and IgG against *C. pneumoniae* have more severe disease with increased airway obstruction, higher doses of ICS, more signs of air trapping and less type-2 inflammation.

## Introduction

Asthma is an inflammatory disease of the airways that is characterized by airway hyperresponsiveness towards various environmental factors. Previous studies suggest that the respiratory tract microbiome may underlie the development of asthma [1] and clinical studies report that up to 45% of adult-onset asthma starts after acute respiratory illness [2, 3].

*Chlamydia pneumoniae* and *Mycoplasma pneumoniae* are intracellular and extracellular bacteria respectively, both causing respiratory tract infection. Chlamydia and *Mycoplasma pneumoniae* have been implicated in the pathogenesis [4] of asthma [5–7], especially adult onset asthma [8]. *Chlamydia pneumoniae* is thought to promote asthma by inducing both airway eosinophilia and neutrophilia with concomitant enhancement of the severity of airways disease [9] and asthma symptoms [10]. Failure to eradicate *C. pneumoniae* can lead to chronic infection, where *C. pneumoniae* enters a state of “latency” in which it is viable but dormant and does not multiply. It however continues to synthetize heat shock protein 60 [11], able to induce a strong inflammatory response and may have intermittent periods of replication. In a study looking at asthmatics and non-asthmatic controls, C. pneumoniae led to an increase of IgE, IL-4 and IFN-gamma in asthmatics as compared to non-asthmatics [12]. *Chlamydia pneumoniae* has also been shown to induce secretion of TNF-alpha and IL-8 [13]. It has been suspected to contribute to airway remodeling [14] by inducing the production of IL-6, IFN-Beta and MMPs that can promote smooth muscle cell proliferation [15]. Moreover, *Chlamydia pneumoniae* seems to be able to impair apoptosis of infected cells leading to chronic infection and to induce ciliostasis in the bronchi. On the other hand, *Mycoplasma pneumoniae* attaches to and destroys ciliated epithelial cells of the respiratory tract mucosa. It induces the secretion of IL-8 and TNF-alpha in vitro [16], Type-2 allergic inflammation in sensitized mice [17] and increased serum IL-4, IL-5 [18] and IgE levels in vivo [19].

As atypical bacterial infection of epithelial cells induces the production of a cascade of cytokines that recruit and activate immune cells, we wanted to evaluate if patients with signs of chronic infection had different inflammatory phenotypes and severity profile.

## Methods

### Subject characteristics

We conducted a prospective study on a series of 406 patients with asthma recruited from the University Asthma Clinic of Liege between 7th of June 2016 and 26th of June 2018. The patients came from routine practice to University Hospital and were recruited by two clinicians involved in asthma. Entry criteria were any patients with asthma who accepted to undergo detailed investigation at the Asthma Clinic. Their demographic, functional and inflammatory characteristics are summarised in Tables 1, 2 and 3.

**Table 1:** Demographic characteristics of asthmatics according to the presence of IgG and IgA against Chlamydia pnaumoniae

	Chlamydia pn IgA−, IgG−	Chlamydia pn IgA−, IgG +	Chlamydia pn IgA + , IgG +
N	145 (35.5%)	193 (47.5%)	68 (17%)
Gender (F, %)	68	63	49*^##^
Age (years)	47 ± 16	51 ± 17*	57.5 ± 13***^#^
Age of onset (years)	34 ± 22	35 ± 22	39 ± 22
Asthma duration (years)	8 (1–23)	10 (3–26)	14.5 (2.5–25)*
BMI (Kg/m2)	26.5 (22.6–29.4)	27 (23.4–30.9)	26.4 (23.3–30)
Tobacco			
Smokers (%, PY)	12.4 (13 (0.3–60)	18.1 (21 (1.4–58)	19.1 (25 (4–68)
Ex-smokers (%, PY)	27.6 (9 (0.2–75)	25.9 (16 (0.8–83)	35.3 (22 (0.8–75)
Non-smokers (%)	60	56	46 *
Environment			
City (%)	24	23	27
Country side (%)	38	44	47
Suburban (%)	30	26	22
Atopy (Y, %)	50	50	49
Exacerbations (n)	0.88 ± 1.70	0.86 ± 1.60	1.06 ± 2.04
Classification			
Mild (%)	56	55	38*^#^
Moderate (%)	16	19	29*
Severe (%)	28	26	31
Dose of ICS (equivalent of beclomethasone)	760 ± 825	795 ± 1034	1022 ± 1042*
Oral corticoids (Y, %)	10	11	10
Biologics (Y, %)	5	3	0
Anti-IgE (n)	1	2	0
Anti-IL5 (n)	4	2	0
Long-term macrolides (Y, %)	2	0.5	3
Antileukotrienes (Y, %)	23	29	29
ACT	15.5 ± 5.2	15.5 ± 5.6	14.5 ± 0.7
ACQ	1.88 ± 1.16	1.99 ± 1.30	2.19 ± 1.13
AQLQ	4.61 ± 1.41	4.52 ± 1.48	4.29 ± 1.28

Results are presented as mean + SD for categorial variables and median (IQR) for skewed distribution. *:  Significantly different from IgA-IgG-group

*: Different from IgA−/IgG−. #: Different from IgA− IgG +

^*^p < 0.05

^**^p < 0.001

^***^p < 0.0001

^#^p < 0.05

^##^p < 0.001

^###^p < 0.0001

**Table 2:** Inflammatory characteristics of asthmatics according to the presence of IgG and IgA against *Chlamydia pneumonia*

	Chlamydia pn IgA−, IgG−	Chlamydia pn IgA−, IgG +	Chlamydia pn IgA + , IgG +
N	145 (35.5%)	193 (47.5%)	68 (17%)
FeNO (ppb)	23 (16–47)	22 (12–38)	21 (14.3–34)
Blood eosino, %	2.7 (1.5–4.4)	2.5 (1.3–4.5)	2.9 (1.7–4)
Blood eosino, /mm^3^	187 (104–399)	181 (92–330)	191.5 (107–272)
Blood neutro, %	55.8 (49.4–62.2)	56 (48.2–62.1)	55.1 (49.2–62.1)
Blood neutro, /mm^3^	3881 (3291–4865)	3931 (3028–5358)	3959 (3162–4995)
Blood mono, %	7.1 (5.9–8.1)	7.7 (6.4–9.2)*	7.2 (5.8–9)
Blood mono, /mm^3^	492 (390–647)	572 (444–707)*	514 (401–644)
Blood lympho, %	33 (27.7–38.4)	32.2 (25.7–39.2)	32.6 (25.9–41.1)
Blood lympho, /mm^3^	2360 (1878–2839)	2298 (1888–2787)	2201 (1780–2777)
IgE (U/L)	74 (28–259.5)	97.5 (26.5–315)	85.5 (28–284)
Fibrinogen, g/l	3.28 (2.66–3.81)	3.32 (2.76–3.84)	3.5 (3.03–3.98)*
CRP, mg/l	2.14 (0.84–5.39)	2.53 (0.99–5.14)	2.47 (1.07–4.86)
Alpha-1-antitrypsin	1.37 (1.21–1.54)	1.43 (1.29–1.58)*	1.47 (1.31–1.58)
Cortisol, nmol/l	175 (138–295)	187 (146–271)	183 (125–268)
Sputum eosino, %	2.1 (0.35–9.6)	1.2 (0.2–4.9)	0.8 (0–3.5)*
Sputum eosino, × 10^3^/gr	45 (2.1–170.3)	16 (0.74–116)	12.1 (0–64.4)*
Sputum neutro, %	61 (44.5–80.4)	63.6 (42.2–83.6)	69.9 (35.5–83.8)
Sputum neutro, × 10^3^/gr	906 (350–2405)	878 (304–3543)	811.5 (294–2292)
Sputum lympho, %	1 (0.2–1.95)	1 (0.2–2)	0.8 (0–1.9)
Sputum lympho, × 10^3^/gr	11.8 (1.65–37.7)	14.4 (2–47.3)	10.1 (0–36.7)
Sputum macro, %	20.5 (10.6–35.4)	20.3 (9.7–36.5)	17.6 (10.3–36.6)
Sputum macro, × 10^3^/gr	311 (123–665)	363 (139–839)	263 (115–846)

Results are presented as mean + SD for categorial variables and median (IQR) for skewed distribution. *:  Significantly different from IgA−IgG−group

*: Different from IgA−/IgG−. #: different from IgA−IgG +

^*^p < 0.05

^**^p < 0.001

^***^p < 0.0001

# p < 0.05

## p < 0.001

### p < 0.0001

**Table 3:** Functional characteristics of asthmatics according to the presence of IgG and IgA against *Chlamydia pneumoniae*

	Chlamydia pn IgA−, IgG−	Chlamydia pn IgA−, IgG +	Chlamydia pn IgA + , IgG +
N	145 (35.5%)	193 (47.5%)	68 (17%)
Post-BD FEV1, %	91 ± 22	90 ± 21	88 ± 23
Reversibility, %	7 ± 12	6 ± 10	6 ± 9
FEV1/FVC, %	79 ± 11	78 ± 10	77 ± 12*
PC20M (mg/ml)	2.81 ± 5.70	2.75 ± 8 .02	2.10 ± 6.32
RV/TLC, %	42 ± 12	45 ± 12	48 ± 10*^#^
DLCO, %	79 ± 16	77 ± 18	75 ± 18
KCO	92 ± 20	90 ± 18	91 ± 20
sGaw (L/sec/kPa/L)	0.82 ± 0.37	0.81 ± 0.42	0.70 ± 0.31*
TLC, %	98 ± 15	97 ± 13	97 ± 14
FRC, %	124 ± 30	124 ± 27	130 ± 28

Results are presented as mean + SD for categorial variables and median (IQR) for skewed distribution. Geometric mean for PC20M. *:  Significantly different from IgA−IgG−group

*: Different from IgA−/IgG−. #: Different from IgA−IgG +

^*^p < 0.05

^**^p < 0.001

^***^p < 0.0001

# p < 0.05

## p < 0.001

### p < 0.0001

Asthma was diagnosed based on the presence of chronic respiratory symptoms such as cough, breathlessness or dyspnoea together with the demonstration of airflow variability. The latter was defined by airway hyper-responsiveness shown by one or more of the following: increase in Forced Expiratory Volume in 1 s (FEV_1_) of > 12% and 200 ml following inhalation of 400 µg salbutamol or inhaled concentration of methacholine provoking a 20% fall in FEV_1_ of < 16 mg/ml. Methacholine challenge was performed according to a standardised methodology as previously described. Subjects were characterised as atopic if they had at least one positive specific IgE (> 0.35 kU/l; Phadia) for at least one common aeroallergen (cat, dog, house dust mites, grass pollen, tree pollen and a mixture of moulds). Exacerbation in the previous year was defined by a course of oral corticoids for at least 3 days in case of asthma worsening. Patients experiencing an exacerbation treated with OCS or antibiotics during the last 6 weeks were excluded.

### Study design

Patients underwent FeNO measurement at a flow rate of 50 ml/s according to the ERS/ATS recommendations (NIOX, Aerocrine, Sweden). FeNO was first measured and followed by spirometry with bronchodilation, sputum induction and blood sampling. All tests were performed on the same day.

Quality of life was assessed using the self-administered Asthma Quality of Life Questionnaire (AQLQ) [20] and asthma control by the Juniper Asthma Control Questionnaire (ACQ) [21] and Asthma Control Test [22].

Sputum was induced and processed as previously reported and was successful in 78% of the patients encountered in our asthma clinic which is similar to previous report. Cell count were estimated on samples centrifuged (Cytospin) and stained with Diff Quick after counting 500 cells (Dade, Brussels, Belgium).

This study was conducted with the approval of the ethics committee of CHU Liege.

### Determination of *Chlamydia pneumoniae* and *Mycoplasma pneumoniae* status

Due to the difficulties of detecting the bacteria in airway secretions, we used indirect methods to differentiate acute from possible chronic infection. Serum *C. pneumonia*-specific antibodies were detected with the aid of a quantitative ELISA-based assay (Savyon® kit) and processed with the Eti-max 3000® analyzer (DiaSorin). Values of IgA greater than or equal to 2 UA/ml were considered positive in the present study. For IgG, a cut-off of ≥ 28 UA/ml was used. The kinetics of IgG and IgA secretion are taken into account when determining acute or chronic infection. Chronic infection diagnosis requires the detection of persistent serum IgG levels and persistence of elevated IgA levels on two samples taken at 2 weeks interval [23]. Patients were then separated in 3 different groups according to their serological results (IgA negative and IgG negative, IgA negative and IgG positive, and IgA positive and IgG positive). Asthmatics who were IgA positive and IgG negative (n = 4) were excluded due to the acute character of respiratory infection.

Serum *Mycoplasma pneumoniae* antibodies were analyzed with a quantitative assay by immunoluminometric method, LIAISON®XL analyzer (DiaSorin). IgM were either positive or negative and IgG were considered positive when ≥ 11 UA/ml.

### Effect of macrolides

14 asthmatics with IgA + /IgG + for *Chlamydia pneumoniae* received macrolides after the first visit at the Asthma Clinic due to the presence of neutrophilic asthma (sputum neutrophil count ≥ 76%) insufficiently controlled with ICS/LABA treatment. Demographic, functional and inflammatory characteristics of these patients were compared before and after treatment with antibiotics.

### Statistical analyses

The results were expressed as mean ± SD or mean ± SEM for continuous variables; median and interquartile ranges (IQR) were preferred for skewed distributions. For categorical variables, the number of observations and percentages were given in each category. Comparisons between different subgroups were performed with a Kruskal–Wallis test. The Spearman correlation coefficient was used to measure the association between clinical parameters. Paired T-test were used to compare patients before and after treatment with macrolides. Variables independently associated to Positivity to Chlamydia (IgA−/IgG + and IgA + /IgG +) were identified by logistic regression. Independent variables such as Age, BMI, Gender, Tobacco status, disease duration, post-BD FEV_1_, post-BD FEV_1_/FVC, RV/TLC, sGaw, ACQ score, AQLQ score, exacerbations during the last year, FeNO, Sputum Eosinophils (%), Sputum neutrophils (%), Blood eosinophils (/mm^3^), Blood neutrophils (/mm^3^), total IgE, and ICS dose were included in the univariate model. Positivity to Chlamydia (IgA−/IgG + and IgA + /IgG +) was used as the dependent variable. A multivariable stepwise forward analysis was done including all independent variables. A p value < 0.05 was considered statistically significant. Statistical analysis was done using STATA version 14.0 (Statistical Software, College Station, TX: StataCorp LP).

## Results

406 asthmatics who underwent a visit to the asthma clinic in stable state with a measurement of serum IgA and IgG for *Chlamydia pneumonia*e and IgG and IgM for *Mycoplasma pneumoniae* were included in this study.

**Fig. 1:**
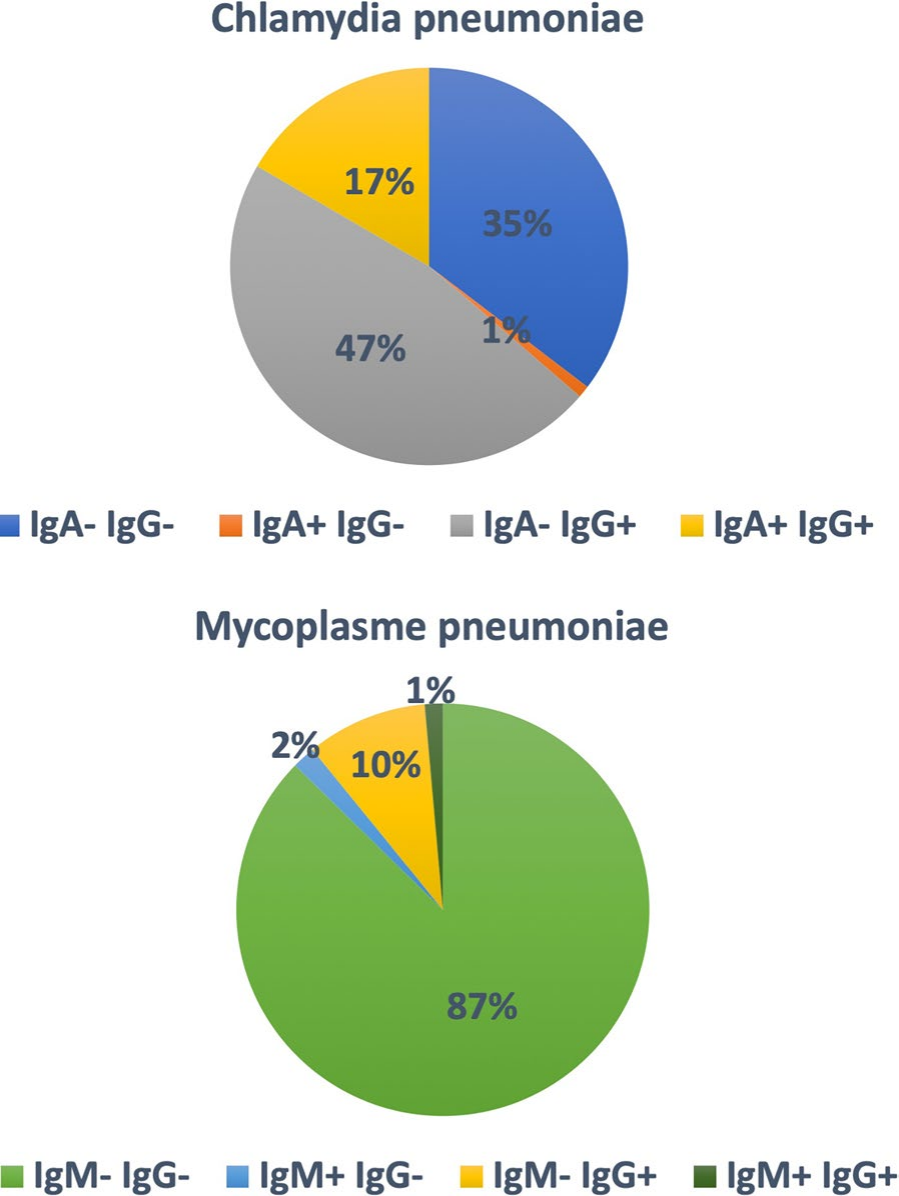
Prevalence of positive IgA/IgG against *Chlamydia pneumoniae* (upper panel) and IgM/IgG against *Mycoplasma pneumoniae* (lower panel)

Only 12.6% of our asthmatic population had IgM and/or IgG against *Mycoplasma pneumoniae* while 65% had IgA and/or IgG against *Chlamydia pneumoniae* (Fig. 1). Seroprevalence for *C. pneumoniae* increased with age, with antibodies detected in 50% of teenagers, in 62% of middle-aged adults and in 70% after 60 years of age. Seroprevalence for *M. pneumoniae* was high in children and teenagers then relatively stable across all other ages, reaching 16% in old age. Due to the low frequency of positive serology against *Mycoplasma pneumoniae*, we did not perform between subgroups statistics as it would have been difficult to draw any conclusions. Looking at *Chlamydia pneumoniae* history of infection, we compared three groups of patients classified according to the serological status. The first group included asthmatics without history of *Chlamydia pneumoniae* infection (IgG and IgA negative) and was used as the control group. The second group comprised of patients with a previous infection with *Chlamydia pneumoniae* (IgG positive but IgA negative). The third group involved asthmatics with chronic infection with *Chlamydia pneumoniae* (IgG and IgA positive).

### Demographic, functional and inflammatory characteristics

#### Asthmatics with *Chlamydia pneumoniae* IgA + /IgG + serology

**Fig. 2:**
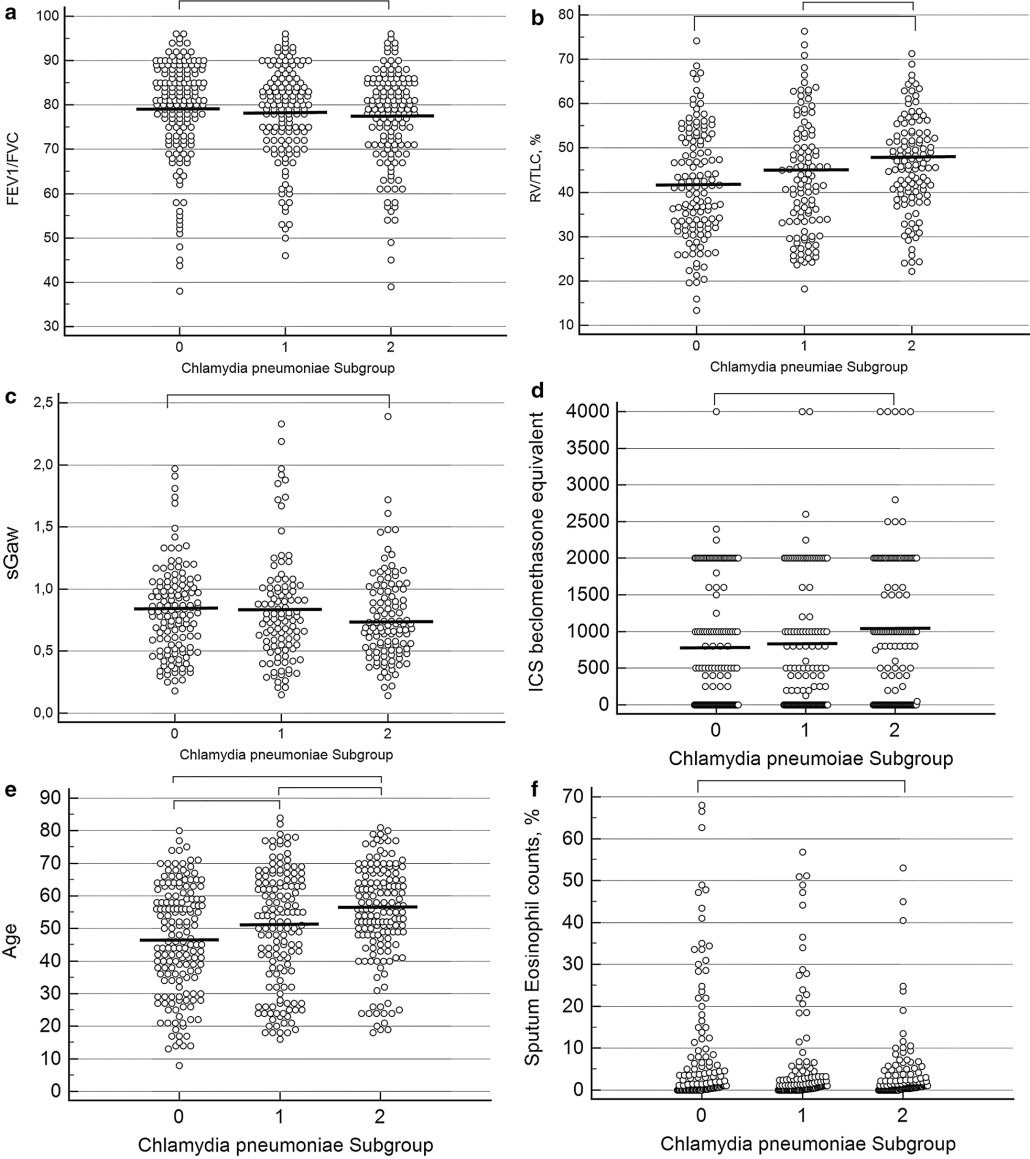
Comparison of patients with *Chlamydia pneumoniae* IgA−/IgG−, IgA−/IgG + and IgA + /IgG + sero-status. Group 0: *Chlamydia pneumoniae* IgA−/IgG−. Group 1: *Chlamydia pneumoniae* IgA−/IgG + . Group 2: *Chlamydia pneumoniae* IgA + /IgG +

Patients with chronic infection to *Chlamydia pneumoniae* were more often males, were older with a lower proportion of non-smokers. Most of them had moderate or severe asthma and were treated with higher doses of inhaled corticosteroids. They had a longer disease duration as compared to asthmatics without a history of *Chlamydia pneumoniae* infection. They also had lower FEV_1_/FVC ratio and more signs of air trapping with a higher residual volume on total lung capacity ratio. Their conductance was lower and they exhibited higher fibrinogen but similar C reactive protein (CRP) levels. We also found lower rates of sputum eosinophilic inflammation in these patients as compared to *Chlamydia pneumoniae* IgA−/IgG− asthmatics (Fig. 2).

We performed a stepwise multivariate regression analysis to highlights predictors of positive serology against *Chlamydia pneumoniae* (Table 4). Predictors of seropositivity to *Chlamydia pneumoniae* were age (OR 1.02, p = 0.001), gender (OR: 0.59, p = 0.015) and FeNO (OR: 0.76, p = 0.019).

**Table 4:** Multivariable stepwise forward analysis

Chlamydia	Total population					
	Univariate			Multivariable		
	OR	95%CI	P-value	OR	95%CI	P-value
Age	1.02	1.01–1.03	< 0.0001	1.02	1.00–1.03	0.001
Gender (M)	0.69	0.46–1.03	0.067	0.59	0.38–0.90	0.015
FENO	0.79	0.64–0.99	0.040	0.76	0.60–0.96	0.019

Variables independently associated to positivity to Chlamydia (IgA−/IgG + and IgA + /IgG +)

### Asthmatics with *Chlamydia pneumoniae* IgA−/IgG + serology

Patients with positive IgG but negative IgA for *Chlamydia pneumoniae* had similar demographic, clinical, functional and inflammatory characteristics as compared to those without a history of *Chlamydia pneumoniae* infection with the only differences being that they were older and had higher blood monocyte counts and alpha-1 antitrypsin levels. It should be noted that both monocytes and alpha-1-antitrypsin levels were within the normal range.

### Asthmatics with positive serology towards *Mycoplasma pneumoniae*

Compared to the other groups, patients with chronic *Mycoplasma pneumoniae* infection were more often males. This was the only significant difference as compared to asthmatics without history (IgM−/IgG−) or with previous infection (IgM−/IgG +) to *Mycoplasma pneumoniae*.

### Association between IgG antibodies levels to *Chlamydia pneumoniae* and demographic, functional and inflammatory characteristics of asthmatics

The median IgG titer for patients with a history of *Chlamydia pneumoniae* infection was 84 (median 30 to 500) while IgG index was 20 (0–22) for the negative counterpart. We found a positive correlation between the level of IgG against *Chlamydia pneumoniae* and age (p = 0.0058, r = 0.14), history of smoking measured in pack-years (p = 0.0043, r = 0.14), air trapping (residual volume on total lung capacity ratio (p = 0.04, r = 0.11)) and alpha-1 antitrypsin level (p = 0.0087, r = 0.14) while there was a negative correlation with FEV_1_/FVC ratio (p = 0.0199, r = − 12), post-BD FEV_1_ (p = 0.04, r = − 0.10) and sGaw (p = 0.04, r = − 0.11).

Looking at IgA levels, we found a positive correlation with age (p = 0.0002, r = 0.18), age of onset (p = 0.02, r = 0.12), history of smoking (in pack years) (p = 0.0052, r = 0.14), the dose of ICS (p = 0.037, r = 0.10), asthma control (ACQ, p = 0.02, r = 0.12), air trapping (RV/TLC, p = 0.006, r = 0.15)) while there was a negative correlation with FEV_1_/FVC (p = 0.04, r = − 0.10) and diffusion capacity (p = 0.047, r = − 0.11).

### Effect of macrolides in a sub-population with sputum neutrophilic inflammation

14 patients received macrolides after the first visit at the Asthma Clinic and came back 9 to 12 months later for a follow-up visit. After macrolides were initiated, we found a significant decrease in blood neutrophils (from 5225/mm^3^ (2999–6627) to 2562 (2240–4692), p = 0.016) and a significant reduction in sputum neutrophils (from 85% (80–95) to 67% (29–87), p = 0.047).

## Discussion

Positive Chlamydia serologic test is far more common than positive Mycoplasma serology. Patients with IgA and IgG against *C. pneumoniae* have more severe disease with increased airway obstruction, higher doses of ICS, more signs of air trapping and less type-2 inflammation. As opposed to *Chlamydia pneumoniae* seropositive asthmatics, patients with a history of *Mycoplasma pneumoniae* infection in our study were infrequent and did not exhibit specific features.

### Prevalence of seropositive patients to *Chlamydia pneumoniae* and *Mycoplasma pneumoniae*

In our study, we found that 65% of asthmatics had signs of past exposure and 17% had signs of chronic infection to *Chlamydia pneumoniae*. The results reported in the literature concerning the prevalence of *Chlamydia pneumoniae* infection in patients with asthma are conflicting due to various methods of measurement and IgA and IgG thresholds used. Our results are very close to those of Gencay et al. [23] who found that 63% of asthmatics had signs of past infection (high IgG) while IgA was present in 52% and serological evidence of chronic infection (high IgG and high IgA) was detected in 18.2% of asthmatics. Cook et al. [24] however found only 14.6% of asthmatics with signs of previous infection defined as IgG 64–256 or IgA > 8, and this percentage was higher in severe asthma (35%) [24]. Hahn et al. [6] found higher rates of exposure to *Chlamydia pneumoniae*, with 72% of asthmatics having *Chlamydia pneumoniae* IgA as compared to 44% in the control group while 92% had IgG against *Chlamydia pneumoniae*. The median *Chlamydia pneumoniae* IgG titer reported in our study was similar to the one reported by Pasternack et al. [25].

As previously described in the literature, we found 13% of patients having positive IgM and/or IgG against *Mycoplasma pneumoniae* which is clearly lower than that observed with *Chlamydia pneumoniae*. Using PCR-based assays, prevalence of M. pneumoniae in the lower airway has been reported to be 16.7 to 19.4% in a study looking at 310 asthmatics [26]**.**

As compared to a general population of healthy subjects [27], the prevalence of seropositivity to Chlamydia and *Mycoplasma pneumoniae* followed the same trend with ageing in our population of asthmatics. The proportion of *Chlamydia pneumoniae* positive patients was similar in asthmatics as compared to healthy subjects while the percentage of *Mycoplasma pneumoniae* positive patients was lower in our asthmatic population as compared to that reported by Tuuminen et al. [27] in healthy subjects. These results suggest that asthma is not a risk factor for infections with atypical bacteria.

### Characteristics of asthmatics with *Chlamydia pneumoniae* IgA + /IgG + serology

In our study, patients with high levels of IgA and IgG against *Chlamydia pneumoniae* were more often males, were older and had a higher proportion of patients with a history of smoking. Wark et al. [28] also compared patients with raised to those with not raised *Chlamydia pneumoniae* antibody titer. They however did not find differences in age, gender, smoking status and atopy. However, they used a lower threshold for IgG with a poor sensitivity for classification of patients in different groups. Factors such as male gender, age and smoking have been previously found to favor the establishment of a persistent *Chlamydia pneumoniae* infection [29]. Age and gender were also highlighted as independent predictors of seropositivity to *Chlamydia pneumoniae* in our multivariate analysis. We also found a positive correlation between IgG and IgA titers against *Chlamydia pneumoniae* and age. It is not surprising that IgG titers was correlated with ageing in our study as there is a higher probability to have repeated contacts with the bacteria. The positive correlation between IgA titers and age and age of onset found in our study suggests a higher risk of persistent infection in older patients and maybe a link between *Chlamydia pneumoniae* infection and late onset asthma. Moreover, ageing is associated with changes in immune system and could increase the risk of persistent IgA against *Chlamydia pneumoniae*. Our results show that a higher history of smoking is associated with higher IgA and IgG titers. This might be explained by a decreased bacterial clearance in patients exposed to cigarette smoke. Higher history of smoking could also be an indirect reflect of the link with ageing as pack-years naturally increase with ageing.

Most of our IgA + /IgG + patients had moderate or severe asthma and were treated with higher doses of inhaled corticosteroids. Whether ICS are a cause or a consequence of chronic infection with *Chlamydia pneumoniae* remains to be determined. Higher doses of ICS may indeed modify local immune responses by reducing cytokine production in the lungs by effector T-cells and eosinophilia but may also negatively affect the host’s ability to eradicate intracellular pathogen by downregulating Natural Killer cell activity and IL-12, IL-10 and TGF-Beta production by macrophages. Higher doses of ICS may however also be a consequence of poorer asthma control due to chronic *Chlamydia pneumoniae* infection during which *Chlamydia pneumoniae* enter a state of “latency” but are still able to synthetize heat shock protein 60 [11] able to induce a strong inflammatory response. Previous studies have found contradictory results. Black et al. [30] reported that patients with elevated levels of both IgA and IgG were more likely to require high-dose ICS and Cook et al. [24] found a higher rate of patients with elevated IgG and IgA titers in severe asthma as compared to mild to moderate asthma. Wark et al. [28] however did not find any difference in the dose of ICS in IgA + /IgG + versus IgG−/IgA− patients, but as previously said, this study used lower IgG threshold with a risk of decreased sensitivity for patients’ classification in different subgroups. The interaction between *Chlamydia pneumoniae*, ICS and the host’s immune system in asthma may thus lead to a vicious circle where increasing the dose of ICS decreases local immunity and triggers re-activation of persistent Chlamydia to active forms of the intracellular bacterium. Laitinen et al. [31] indeed reported that corticosteroids could reactivate *Chlamydia pneumoniae* infections in up to 60% of mice. This could explain why severe asthmatics, treated with higher doses of ICS, are more prone to be *Chlamydia pneumoniae* IgA + /IgG + . Looking at IgA levels, we found a positive correlation with the dose of ICS suggesting a higher risk of persistent infection in patients treated with higher doses of ICS. Moreover, elevated IgA levels against *Chlamydia pneumoniae* may be an important marker of subjects who are less able to clear *Chlamydia pneumoniae* [32]. Other previous studies have also reported that asthmatics with earlier *Chlamydia pneumoniae* infection are more likely to develop steroid-resistant asthma, and airway neutrophilia [30, 33].

### Inflammatory and functional characteristics of asthmatics with *Chlamydia pneumoniae* IgA + /IgG + serology

In our study, asthmatics in stable state with chronic *Chlamydia pneumoniae* also displayed lower eosinophilic airway inflammation. Though immune response dysregulation from C. pneumoniae with predominant Type-2 response has been described in the bacterial allergy model for asthma pathogenesis [34], chronic infection with C. pneumoniae in severe asthma has been linked to exaggerated Th1/Th17 responses and neutrophilic inflammation in vitro [35]. Wark et al. [28] found that patients positive for *Chlamydia pneumoniae* had significantly more sputum neutrophils (4.6 × 10^6^ C/mL) and monocytes during acute asthma with *Chlamydia pneumoniae* infection, but no significant differences in sputum cell count was found one month after acute infection between those who had antibodies and those who had not. In our study, no association was found between chronic infection and sputum neutrophil counts. We however observed significantly lower sputum eosinophil counts in patients with a serologic signature of *Chlamydia pneumoniae* infection. It could be suggested that the decrease in sputum eosinophils observed in asthmatics with persistent IgA and IgG against *Chlamydia pneumoniae* might be due to treatment with higher doses of ICS in this subgroup. When focusing on the steroid naïve patients (n = 145) included in our study, we however confirm lower numbers of sputum eosinophils in asthmatics with IgA + /IgG + against *Chlamydia pneumoniae* (0.6 (0–1.6)) as compared to IgA−/IgG− (1.4 (0.4–4.2), p < 0.05). Additionally, no association was found with IgE, as it has been previously reported [12].

In our population, IgA + /IgG + patients with asthma had lower FEV_1_/FVC ratio and sGAW indicating more severe airway obstruction and more signs of air trapping with a higher residual volume on total lung capacity ratio. It might be that the lower FEV_1_/FVC and higher RV/TLC observed in this *Chlamydia pneumoniae* IgA + /IgG + asthmatics is a consequence of the older age of this subgroup. We indeed recently demonstrated that elderly asthmatics had higher RV/TLC and lower FEV_1_/FVC [36]. We found a positive correlation between IgG titers against *Chlamydia pneumoniae* and measure of air trapping while there was a negative correlation with FEV_1_/FVC, post-BD FEV_1_ and sGaw.

Ten Brincke et al. [37] previously showed that patients with IgG antibodies had a fourfold greater decline in post-BD FEV1/FVC ratio as compared with patients without elevated titers of IgG. This suggests that the respiratory pathogen might be involved in airway remodeling. Pasternack et al. found that adults with nonatopic asthma with chronic *Chlamydia pneumoniae* infection had a faster decline in FEV_1_ [25]. We found elevated RV/TLC ratio in IgA + /IgG + patients. A small study conducted by Weiss et al. [38] in a population of children also found signs of elevated trapped air after Chlamydia pneumoniae infection. Our study is the first attempt to look at air trapping in adult IgA + /IgG + patients with asthma in stable state. We found signs of air trapping suggestive of distal airway dysfunction in patients with chronic infection with *Chlamydia pneumoniae*. This might be due to remodeling induced by chronic infection with this intra-cellular bacterium. We are the first to report a decreased conductance in IgA + /IgG + patients with asthma, an indirect sign of increased bronchial hyperresponsiveness.

Even if we found an increased fibrinogen level in the IgA + /IgG + asthmatics, this level remained within the normal range. This may reflect the low grade chronic inflammation induced by the persistence of intracellular *Chlamydia pneumoniae*.

### Asthmatics with serologic signature of *Mycoplasma pneumoniae* infection

As opposed to *Chlamydia pneumoniae* seropositive asthmatics, patients with a history of *Mycoplasma pneumoniae* infection were not frequent in our study and did not exhibit specific features. This is in line with the findings of Ansarin et al. who reported no difference in asthma control and lung function tests between asthmatics with and without evidence of chronic *Mycoplasma pneumoniae* infection [39].

### Effect of macrolides in a sub-population with sputum neutrophilic inflammation

The more frequent type-1 inflammation observed in *Chlamydia pneumoniae* IgA + /IgG + asthmatics has led to consider treatment of atypical infections as an aspect of the management of asthma patients. Recent studies have evaluated the benefits of using macrolides in asthma and have shown improvement in symptoms and reduction of exacerbations [40]. In patients serologically positive for atypical bacteria, improved lung function [41] and asthma control [42] have been demonstrated with macrolides.

Macrolide mechanisms of action in asthma are thought to be directly anti-inflammatory or indirectly anti-inflammatory with an anti-microbial against chronic atypical infections [10]. A pre-existing chronic *Chlamydia pneumoniae* lung infections could indeed explain the effect of macrolides in asthma [43]. Kraft et al. [41] reported clinical improvements with clarithromycin in asthmatics PCR-positive for *Chlamydia pneumoniae* and *Mycoplasma pneumoniae* but this was not confirmed by other groups [42, 44].

In our study, 14 patients IgA and IgG positive against *Chlamydia pneumoniae* received macrolides after the first visit at the Asthma Clinic for neutrophilic asthma insufficiently controlled with ICS/LABA treatment. Patients came back 9 to 12 months later for a follow-up visit. We found a significant decrease in blood neutrophils and a significant reduction in sputum neutrophils after treatment with macrolides. Previous trials have found an improvement in FEV_1_ value after antibiotic treatment. Like the study of Gibson et al.[45], we did not find a significant change in lung function tests after treatment in our small cohort. We found a reduction in exacerbation rate after treatment with macrolides that however remained nonsignificant (0.33 ± 0.5 versus 0.11 ± 0.33, p = 0.34). Previous studies have suggested a beneficial effect of macrolides in neutrophilic asthma [40, 45]. In our study focusing on hyperneutrophilic patients, we found a reduction in sputum neutrophils from 85 to 67% (p < 0.05). Gibson did not find a significant reduction in sputum neutrophils but median neutrophil count was 34% in patients receiving azithromycin. Our study goes further, indicating that treating patients with *Chlamydia pneumoniae* IgA + /IgG + and sputum neutrophils ≥ 76% with macrolides may allow a normalization of bronchial neutrophilic inflammation. Concerns about adverse effects of macrolides however include development of antibiotic resistance.

### Study limitation

A potential limitation of the present study is that infection with *Chlamydia pneumoniae* and *Mycoplasma pneumoniae* have been inferred serologically without isolation of organism. *Chlamydia pneumoniae* and *Mycoplasma pneumoniae* are however difficult to culture. We used IgA and IgG measurement for identification of chronic infection without the detection of *Chlamydia pneumoniae* mRNA within tissue and cell types by PCR. Direct methods such as mRNA detection can establish the presence of the pathogen and perhaps its viability. The sensitivity is however limited due to low copy numbers or sampling problems due to deep tissue intracellular location for this species. Therefore seroreactivity is valuable to differentiate acute from chronic infection.

## Conclusion

Chlamydia serologic test is more often positive than Mycoplasma serology in asthmatics. Patients with elevated levels of IgA and IgG against *C. pneumoniae* have more severe disease with increased airway obstruction, higher doses of ICS, more signs of air trapping and less type-2 inflammation. As opposed to *Chlamydia pneumoniae* seropositive asthmatics, patients with a history of *Mycoplasma pneumoniae* infection did not exhibit specific features. Treatment of *Chlamydia pneumoniae* seropositive neutrophilic asthmatics with macrolides significantly decrease sputum and blood neutrophils.

## Data Availability

Data are available upon reasonable request.

## Abbreviations

ACQ: Asthma control questionnaire
ACT: Asthma control test
AQLQ: Asthma quality of life questionnaire
BMI: Body mass index
*C. pneumoniae*: *Chlamydia pneumoniae*
CRP: C-reactive protein
DLCO: Diffusion capacity of lung for carbon monoxyde
Eosino: Eosinophil
FeNO: Fractional exhaled nitric oxide
FEV_1_: Forced expiratory volume in one second
FRC: Functional residual capacity
FVC: Forced vital capacity
ICS: Inhaled corticosteroids
IFN: Interferon
IgA: Immunoglobin A
IgE: Immunoglobin E
IgM: Immunoglobin M
IgG: Immunoglobin G
IL: Interleukin
IQR: Interquartile range
KCO: Transfer coefficient of the lung for carbon monoxide
Lympho: Lymphocyte
Macro: Macrophage
MMP: Matrix metalloproteinase
Mono: Monocyte
*M. pneumoniae*: *Mycoplasma pneumoniae*
Neutro: Neutrophil
OCS: Oral corticosteroids
PC20: Methacholine concentration provoking a 20% fall in FEV_1_
RV: Residual volume
SD: Standard deviation
sGaw: Specific airway conductance
TLC: Total lung capacity
TNF: Tumor necrosis factor
